# A cross-sectional study on health behavior changes during COVID-19 among adults in Malaysia

**DOI:** 10.3389/fpubh.2024.1465996

**Published:** 2024-10-28

**Authors:** Norbaidurah Ithnain, Rosnani Kassim, Nadia Amirudin, Siti Nurhanim Mohamed Aimanan, Manimaran Krishnan, Albeny Joslyn Panting

**Affiliations:** ^1^Institute for Health Behavioural Research, Ministry of Health, Setia Alam, Malaysia; ^2^Faculty of Social Sciences and Humanities, Open University Malaysia, Kuala Lumpur, Malaysia

**Keywords:** health behaviors, COVID-19, Malaysia, eating behaviors, physical activity, smoking, alcohol, sleep patterns

## Abstract

**Introduction:**

COVID-19 has triggered significant disruptions globally, necessitating swift adaptations in individuals’ health behaviors.

**Methods:**

This cross-sectional study was conducted during Phase Four of Malaysia’s National Recovery Plan and examines how the pandemic has affected health behaviors among adult Malaysians. The study gathered data online using convenience sampling with 1,004 respondents aged 18 and above. The research focused on diverse health domains, including eating habits, physical activity, smoking, alcohol consumption, and sleep patterns. The Wilcoxon Signed Rank test and descriptive statistics were employed to compare health behaviors before and after COVID-19.

**Results:**

Findings indicate noteworthy shifts in eating behaviors, with increased water and fruit consumption (*p* < .001). The frequency of home-cooked meals stayed relatively stable despite declining dinner preference and increasing daily snacks. Physical activity declined, marked by increased sedentary behavior and screen time (*p* < .001). There were differences in the patterns of smoking and alcohol consumption; some had started these behaviors during the pandemic. Notably, intentions to quit smoking among respondents were more prominent than attempts to stop drinking. Respondents’ sleep patterns also changed, with more sleeping fewer than seven hours daily (*p* < .001).

**Conclusion:**

The study emphasizes the need for focused interventions to address new challenges by highlighting the impact on health behaviors. As Malaysia navigates the post-pandemic landscape, understanding and mitigating the persisting effects on health behaviors are crucial for promoting overall well-being.

## Introduction

In late 2019, the highly contagious COVID-19 virus emerged, quickly spreading to 114 countries, territories, and places worldwide, causing an unparalleled global health emergency. By March 2020, the World Health Organization (WHO) declared the outbreak a pandemic. Numerous nations implemented lockdowns and mobility restrictions to curb the spread of the virus. As of September 2024, there have been almost 6 million deaths worldwide. In Malaysia, reports indicated approximately 5.3 million COVID-19 infections, with an estimated 36,652 deaths attributed to the virus (1).

Although the virus has continued to spread, many aspects of life have gradually improved since the onset of the pandemic. One notable advancement is the significant decrease in the risk of severe disease and death due to the introduction of vaccinations and boosters. Immunization has allowed more Malaysians to return to their regular lives over the past year. However, the COVID-19 pandemic has resulted in substantial adverse health, economic, and psychological effects that continue to affect the community, even as many of the pandemic’s restrictions have been loosened and transmission rates have declined.

At the beginning of the pandemic, Malaysia, like many other countries, implemented the Movement Control Order (MCO) to restrict social contact and physical movement to lower the spread of the virus among the broader public. However, COVID-19-related preventative measures and restrictions (such as confinement) were shown to negatively impact the health and well-being of the adult population (2). Additionally, it is particularly concerning when individuals engage in unhealthy behaviors, given the potential influence on COVID-19-related risks and consequences (3).

Previous research suggested that during the COVID-19 lockdown, lower psychological well-being and mental health were linked to the adoption of unhealthy lifestyle habits, such as using tobacco products (2), eating an unhealthy diet (4, 5), getting less sleep (4), and not exercising (6). These habits can contribute to mental health issues, including post-traumatic stress disorder (PTSD) and other stress-related conditions (2). Additionally, they can lead to metabolic disorders, such as overweight and obesity, which are associated with a range of health complications.

Despite the ongoing presence of COVID-19, individuals must adapt to coexisting with the virus. Many people remain at heightened risk of engaging in unhealthy behaviors. Therefore, this study compares health behaviors among adult Malaysians before and during the COVID-19 pandemic in the following domains: eating habits, physical activity, smoking, alcohol consumption, and sleep patterns. The study’s results will show how adult Malaysians’ health behaviors changed before and during the COVID-19 pandemic.

## Method

### Study design

This cross-sectional study was conducted from January to March 2022, during Phase Four of the National Recovery Plan (PPN), focusing on Malaysian citizens’ health and health-related behaviors. The minimum sample size required for this study was 385 respondents, calculated using a power analysis based on a 95% confidence level (5). This calculation assumed that the sample proportion would be within 0.05 of the population proportion, which was estimated to be 0.50, reflecting a population size of 29.1 million Malaysians (Department of Statistics, Malaysia, DOSM 2020). To ensure robust results and account for potential non-responses or incomplete surveys, we added a 30% contingency to the initial sample size. As a result, we targeted a minimum of 500 respondents for the study.

A convenience sampling technique was employed to recruit potential study respondents through various online platforms, including Facebook, Twitter, Instagram, WhatsApp, and some internet websites. WhatsApp was the most used platform, with 88% of respondents using it at least once a week. During the COVID-19 pandemic, social media platforms, especially Facebook, are an effective and cost-efficient research strategy for collecting large-scale nationwide surveys (7).

### Data collection procedure

An anonymous online questionnaire was created using Google Forms, allowing respondents to fill in without signing in. The form provided information about the study and required informed consent. Respondents could choose to agree or disagree, and they could withdraw midway. The survey was anonymous, preserving privacy by not collecting personal identifying information. Data was stored for 5 years before being destroyed. Data was entered into an offline document after being retrieved from the Google Form and then destroyed. Participants were selected based on the following inclusion criteria: Malaysian citizens aged 18 years and above, capable of reading and writing in Malay, and proficient in using computers and mobile devices. Exclusion criteria included non-Malaysian citizens, individuals under 18 years of age, and those unable to understand the survey instructions.

### Study instrument

The questionnaire used in this study comprised questions regarding health behaviors both before and during the pandemic, organized into five categories: (a) sociodemographic information, (b) eating behavior, (c) physical activity, (d) alcohol and tobacco use, and (e) sleep quality. Respondents were asked to indicate whether their behaviors had improved, worsened, or remained the same since the onset of the pandemic. These responses were based on the concept of “Self-Perceived Changes” to ascertain variations in health behavior before and during COVID-19 (2, 4–6). The questionnaire was developed in Malay, the national language.

To ensure the questionnaire’s validity and reliability, it underwent a review process by several experts, who provided recommendations for specific changes. Additionally, a hard copy of the questionnaire was pretested with 10 respondents to assess the clarity of the wording and the average time required for completion. The finalized version was then converted into an online format using Google Forms. Subsequently, a pilot study involving 30 respondents was conducted to evaluate the online survey’s formatting and the instrument’s reliability. The reliability scores from the pilot study indicated good consistency, with scores of 0.716 for items related to family relationships and 0.914 for those concerning psychological health. Overall, this rigorous process of development, expert review, pretesting, and pilot study ensured that the questionnaire was both appropriate and effective for the objectives of this research.

### Ethical approval and consent form

The Medical Review & Ethics Committee (MREC) granted ethical approval for this study (NMRR-21-1866-61534). All respondents were informed of the study’s purpose, potential risks, and benefits before the start of the survey. Additionally, they were free to leave the study at any time. The informed consent form will be signed by those who consent to participate.

### Data analysis

All completed Google Form questionnaires were downloaded into a Microsoft Excel sheet for data editing, sorting, and coding. The SPSS Statistical Package Programme Version 20.0 for Windows (SPSS Inc., Chicago, IL, USA) was then used to analyze the data. Next, the Wilcoxon Signed Rank test and descriptive statistics were employed to compare health behaviors before and after COVID-19.

## Results

### Demographic profile of the respondents

The online survey received 1,004 responses from Malaysia’s 16 states and federal territories. The majority of them were females (69.0%), Malays (74.4%), between the ages of 36 and 45 (38.9%), had a tertiary education (79.2%) and worked in government (83.3%). Table 1 shows that 35.5% of the respondents earned between RM 4001 and 8,000 monthly.

**Table 1:** Demographic characteristics (*n* = 1,004).

Sociodemographic characteristics		*n*	%
State	Johor	38	3.8
	Kedah	67	6.7
	Kelantan	40	4.0
	Melaka	27	2.7
	Negeri Sembilan	27	2.7
	Pahang	53	5.3
	Perak	88	8.8
	Perlis	40	4.0
	Pulau Pinang	61	6.1
	Sabah	106	10.6
	Sarawak	112	11.2
	Selangor	200	19.9
	Terengganu	18	1.8
	Wilayah Kuala Lumpur	60	6.0
	Wilayah Labuan	1	0.1
	Wilayah Putrajaya	66	6.6
Gender	Male	311	31.0
	Female	693	69.0
Age	18–25	35	3.5
	26–35	301	30.0
	36–45	391	38.9
	46–55	208	20.7
	56–59	49	4.9
	60 and above	20	2.0
Ethnicity	Malay	747	74.4
	Chinese	53	5.3
	Indian	27	2.7
	Bumiputra Sabah	92	9.2
	Bumiputra Sarawak	72	7.2
	Others	13	1.3
Religion	Islam	824	82.1
	Buddha	34	3.4
	Hindu	21	2.1
	Kristian	123	12.3
	Others	2	0.2
Education level	Primary	2	0.2
	Secondary	205	20.4
	Higher Education	795	79.2
	No formal education	2	0.2
Household income	Less than RM4,000	350	34.9
	RM 4001–8,000	356	35.5
	More than RM 8001	298	29.7
Occupation	Self-employed	33	3.3
	Government servant	836	83.3
	Private workers	66	6.6
	Student	18	1.8
	Pension	19	1.9

### Changes in health behavior

This section aimed to investigate the changes in health behaviors, including eating habits, physical activity, smoking, alcohol consumption, and sleep quality among Malaysian adults before and during the COVID-19 pandemic.

### Eating behavior

Table 2 presents the significant changes in the respondents’ eating behaviors throughout the pandemic. Although the frequency of eating four to six times a day increased, the reported percentage of people who ate three meals a day decreased slightly from 59.4 to 54.1%; however, this difference was not statistically significant. Dinner remained the least preferred meal, rising marginally from 46.1 to 49.0% (*p* < 0.001) during the pandemic. On the other hand, lunch continued to be the respondents’ main meal, both before and during COVID-19. More importantly, the percentage of respondents who ate their main meals outside their homes three to six times a week increased from 32.1 to 34.7%. This increase is likely because the data was collected during Phase 4 of the National Recovery Plan when all social and economic sectors had resumed operations.

**Table 2:** Comparison of eating behaviors before and during COVID-19.

Eating behaviors		Before COVID-19		During COVID-19		*p*
		*n*	(%)	*n*	(%)	
How many meals did you eat daily?	1 meal a day	17	1.7	31	3.1	0.145
	2 meals a day	215	21.4	248	24.7	
	3 meals a day	596	59.4	543	54.1	
	4 meals a day	138	13.7	132	13.1	
	5 meals a day	31	3.1	34	3.4	
	6 or more meals a day	7	0.7	16	1.6	
Which is your main preferred meal?	Breakfast	203	20.2	206	20.5	0.098
	Lunch	636	63.3	604	60.2	
	Dinner	165	16.4	194	19.3	
Which meal is taken the least**?**	Breakfast	371	37.0	325	32.4	<0.001*
	Lunch	170	16.9	187	18.6	
	Dinner	463	46.1	492	49.0	
How often do you eat home-cooked meals in a week?	Never	24	2.4	36	3.6	0.434
	1–2 times a week	286	28.5	270	26.9	
	3–6 times a week	465	46.3	445	44.3	
	Everyday	229	22.8	253	25.2	
How often do you eat food from outside (order, takeaway, delivery, eat out) in a week?	Never	72	7.2	60	6.0	<0.001*
	1–2 times a week	539	53.7	497	49.5	
	3–6 kali a week	322	32.1	348	34.7	
	Everyday	71	7.1	99	9.9	
Plain water intake per day	Less than 6 glasses	254	25.3	194	19.3	<0.001*
	6–8 glasses	509	50.7	521	51.9	
	More than 8 glasses	241	24.0	289	28.8	
How often in a week do you eat fast food?	Never	109	10.9	129	12.8	0.092
	1–2 times a week	783	78.0	721	71.8	
	3–6 kali a week	107	10.7	145	14.4	
	Everyday	5	0.5	9	0.9	
How often do you eat snacks?	Never	33	3.3	30	3.0	0.003*
	Rarely	745	74.2	712	70.9	
	Always	226	22.5	262	26.1	
Frequency of eating fruits per day	Did not eat	149	14.8	123	12.3	<0.001*
	1 serving a day	587	58.5	576	57.4	
	2 servings a day	195	19.4	224	22.3	
	3 serving or more a day	73	7.3	81	8.1	
Frequency of eating vegetables per day	Did not eat	57	5.7	59	5.9	0.971
	1 serving a day	385	38.3	382	38.0	
	2 servings a day	404	40.2	403	40.1	
	3 serving or more a day	158	15.7	160	15.9	
How do you rate your eating habits?	Not healthy	30	4.9	49	4.9	0.656
	Less healthy	320	30.6	307	30.6	
	Healthy	588	55.9	561	55.9	
	Healthier	66	8.7	87	8.7	

^*^Significant at *p*-value < 0.05 (Wilcoxon Signed Ranks test).

Furthermore, the respondents were more likely to eat snacks during COVID-19 than before the pandemic (22.5 to 26.1%, *p* < 0.001). Additionally, there was a statistically significant increase in the daily consumption of fruits (two servings per day) and plain water (drinking more than eight glasses) from 24.0 and 19.34% to 28.8 and 22.3%, respectively. Conversely, no significant differences were found in the number of vegetables and fast food consumed. This may be because, even before the pandemic, a sizable portion of the population did not consume the recommended number of fruits and vegetables. All these findings should be considered when recommending dietary practices.

### Comparison of physical activity before and during COVID-19

Table 3 compares the respondents’ physical activity levels before and during COVID-19. All five questions had a significant difference (*p* < 0.001). During the COVID-19 pandemic, there was an increase in respondents who did not engage in any physical activity (17.5 to 29.9%) as well as those who spent more than 4 h of screen time on weekdays and weekends (51.2 to 66.0% and 47.2 to 58.8%, respectively). There was also a slight decrease in those who engaged in 30–120 min of weekly physical activity, from 41.8% before the pandemic to 32.6% during it. Similarly, the proportion of people who did not engage in physical activity increased from 16.2 to 26.4%. Internet use became the most popular activity both before and during the pandemic. Furthermore, the time spent on the Internet increased from 32.4 to 47.0%. Those who engaged in physical activity, on the other hand, fell from 21.7 to 12.0% during the pandemic.

**Table 3:** Comparison of physical activity before and during COVID-19.

Physical activity		Before COVID-19		During COVID-19		*p*
		*n*	(%)	*n*	(%)	
Frequency of physical activity per week	Not doing any	176	17.5	300	29.9	< 0.001*
	1–2 times a week	534	53.2	492	49.0	
	3 times a week	187	18.6	128	12.7	
	4–5 times a week	78	7.8	60	6.0	
	6–7 times a week	29	2.9	24	2.4	
Duration of activity in a week	Not doing any	163	16.2	265	26.4	< 0.001*
	Less than 30 min	350	34.9	374	37.3	
	30–120 min	420	41.8	327	32.6	
	More than 120 min	71	7.1	38	3.8	
Daily screen time (weekdays)	Less than 1 h	24	2.4	17	1.7	< 0.001*
	1–2 h	165	16.4	108	10.8	
	2–4 h	301	30.0	216	21.5	
	More than 4 h	514	51.2	663	66.0	
Daily screen time (weekend)	Less than 1 h	37	3.7	30	3.0	< 0.001*
	1–2 h	154	15.3	109	10.9	
	2–4 h	339	33.8	275	27.4	
	More than 4 h	474	47.2	590	58.8	
Most frequent activity	Internet surfing	325	32.4	472	47.0	< 0.001*
	Exercise	218	21.7	120	12.0	
	Watching television	108	10.8	96	9.6	
	Cooking/doing house chores	201	20.0	186	18.5	
	Gardening	38	3.8	37	3.7	
	Others	114	11.4	93	9.3	

*Significant at *p*-value < 0.05 (Wilcoxon Signed Ranks test).

### Self-perceived changes in physical activity and screen time due to COVID-19

This study revealed that 35.5% of respondents reported decreased physical activity for various reasons, including being concerned about the risk of contracting COVID-19, needing to work overtime, or being preoccupied with family and children. Additionally, 43.4% of respondents said they had increased their screen time, with the most common reasons cited being work, boredom, and entertainment. Because of the COVID-19 pandemic, half of the respondents (53.0%) believed they spent more time sitting and lying down (Table 4).

**Table 4:** Self-perceived changes in physical activity and screen time due to COVID-19.

Self-perceived changes	*n*	%
Physical activity changes (*n* = 1,004)		
Increased	141	14.0
Decreased	356	35.5
It has not changed; my physical activity was low before COVID-19, and it is still the same now	231	23.0
It has not changed; my physical activity was moderate before COVID-19, and it is still the same now	242	24.1
It has not changed; my physical activity was high before COVID-19, and it is still the same now	34	3.4
Reason for decreased physical activity (*n* = 356)		
I am worried about the risk of getting infected with COVID-19	137	38.5
I work overtime	73	20.5
I am busy handling family/kids	62	17.4
I do not have exercise equipment	8	2.2
I do not have enough space	24	6.7
I cannot do physical activities alone	23	6.5
Others	29	8.1
Screen time changes (*n* = 1,004)	*n*	%
Increased	436	43.4
Decreased	46	4.6
It has not changed; the amount of screen time I spent was low before COVID-19, and it is still the same now	105	10.5
It has not changed; my screen time was moderate before COVID-19, and it is still the same now	292	29.1
It has not changed; my screen time was high before COVID-19, and it is still the same now	125	12.5
Reason for increased screen time (*n* = 436)	*n*	%
Work	180	41.3
Entertainment	79	18.1
Online learning	33	7.6
Boredom	94	21.6
The need to help with the child’s homework	29	6.7
Others	21	4.8
Perception of whether COVID-19 affected the amount of time spent sitting or lying down	*n*	%
No, no changes at all	364	36.3
Yes, I spent less time sitting or lying down	108	10.8
Yes, I spent more time sitting or lying down	532	53.0

### Smoking and alcohol consumption

Of all the respondents, 77 were smokers during the survey. 3.9% of them stated that COVID-19 was when they first started smoking. Of the smokers who began before the pandemic, half (56.8%) said they had not changed their smoking habits. In comparison, the remaining smokers (21.6%) showed a similar percentage of either an increase or decrease in smoking habits. Furthermore, compared to smokers who merely intended to stop, 32.5% of current smokers (42.9%) had made attempts to quit (Table 5).

**Table 5:** Smoking habits.

Smoking habits		*n*	%
Do you smoke? (*n* = 1,004)	Yes	77	7.7
	No	927	92.0
Smoked since before COVID-19 (*n* = 77)	Yes, I smoked before COVID-19	74	96.1
	No, I have only been smoking since COVID-19	3	3.9
Compared to before COVID-19, have your smoking habits changed? (*n* = 74)	Increased	16	21.6
	No changes	42	56.8
	Decreased	16	21.6
Do you intend to quit smoking during this COVID-19 period? (*n* = 77)	Yes, I have the intention to quit	25	32.5
	Yes, I have the intention and tried to quit	33	42.9
	No, I do not have the intention to quit	19	24.7

Regarding alcohol intake, 93 respondents reported having a current alcohol intake (Table 6). Of these, 10.8% stated that their first drinking experience occurred during COVID-19. Of this group of drinkers, 34.9% reported drinking less, 7.2% reported drinking more, and 57.8% reported no changes in alcohol consumption. Furthermore, most drinkers (61.3%) stated they had no intention of quitting.

**Table 6:** Alcohol consumption status.

		*n*	%
Do you drink alcoholic beverages? (*n* = 1,004)	Never	911	90.7
	Yes, daily	1	0.1
	Yes, 1–2 times a week	12	1.2
	Yes, 3–4 times a week	4	0.4
	Yes, occasionally	76	7.6
Did you drink alcohol before COVID-19 (*n* = 93)	Yes, I drank before COVID-19	83	89.2
	No, I just drank since COVID-19	10	10.8
Compared to before COVID-19, has your alcohol consumption changed? (*n* = 83)	Increased	6	7.2
	No changes	48	57.8
	Decreased	29	34.9
Do you intend to quit alcohol consumption during this COVID-19 period? (*n* = 93)	Yes, I have the intention to quit	18	19.4
	Yes, I have the intention and tried to quit	18	19.4
	No, I do not have the intention to quit	57	61.3

### Sleep pattern

There was a significant difference in sleeping hours before and during the COVID-19 pandemic (*p* < 0.001). Approximately half of the respondents (54.6%) reported sleeping less than 7 hours during the COVID-19 pandemic, compared to 48.1% before the pandemic (Table 7). Most respondents (63.5%) and (32.5%) stated that their bedtime and wake-up times remained the same (Table 8).

**Table 7:** Comparison of sleeping hours before and during COVID-19.

Health behavior		Before COVID-19		During COVID-19		*p*
		*n*	(%)	*n*	(%)	
Sleeping hours per day	Less than 7 h	483	48.1	548	54.6	< 0.001*
	7–9 h	493	49.1	419	41.7	
	More than 9 h	28	2.8	37	3.7	

*Significant at *p*-value < 0.05 (Wilcoxon Signed Ranks test).

**Table 8:** Bedtime and wake time changes compared before COVID-19.

Bedtime	*n*	%	Wake time	*n*	%
Going to bed early	180	17.9	Waking up early	273	27.2
No changes	638	63.5	No changes	628	62.5
Going to bed late	186	18.5	Waking up late	103	10.3

## Discussion

The COVID-19 pandemic has caused numerous changes in people’s health-related behaviors, which provides a unique lens through which to view the dynamics of lifestyle modifications during this historical period. Overall, the findings of this study are crucial due to the significant disruptions caused by the COVID-19 pandemic, which necessitated rapid changes in health behaviors globally. This study, conducted during Phase Four of Malaysia’s National Recovery Plan, sheds light on the pandemic’s impact on Malaysians’ eating habits, physical activity, smoking, alcohol consumption, and sleep patterns. These insights are vital for developing effective public health interventions and policies to address these behavior changes’ immediate and potentially long-lasting effects.

In regards to eating habits, the results of this study demonstrate that during the pandemic, over half of the respondents drank the recommended six to eight glasses of plain water per day, as advised by the Ministry of Health (MOH) Malaysia (8). Additionally, there was an increase in the frequency of the recommended fruit intake, which is two servings per day, and this result was consistent with a study conducted in Hong Kong (4). Some people ate more fruits during the pandemic to bolster their immune systems (9). On the other hand, the psychological impact of the pandemic, including stress coping mechanisms, may contribute to these changes, as individuals may resort to eating as a means of alleviating stress. The psychological impact of the pandemic may have influenced changes in dietary habits, with some individuals increasing their fruit intake as a potential coping mechanism for stress. While this shift could be viewed positively, it is important to consider that nearly 80% of respondents still consumed fewer than three servings of vegetables daily, indicating a consistent low intake of vegetables both before and during the pandemic. This aligns with national data showing that 94.9% of Malaysians do not meet the recommended vegetable intake (10). Additionally, although there was an increase in snack consumption, this finding alone does not imply negative dietary changes, as it depends on the type and quantity of snacks consumed. Overall, while fruit intake may have increased, the persistent low vegetable consumption remains a concern (10).

The findings of this study also indicate a concerning decrease in physical activity during the pandemic, accompanied by a rise in sedentary behaviors and screen time, aligning with findings from other studies (5, 11). Given the well-established links between physical inactivity and adverse health outcomes, this shift raises significant public health considerations (12). According to a review of 21 studies about the impact of physical activity on mental health during COVID-19, people who regularly engaged in physical activity (high volume and frequency) and adhered to a regular schedule of physical activity reported fewer symptoms of anxiety and depression (13). The study also emphasizes the impact of increased screen time on work, boredom, and entertainment, reflecting the broader trend of heightened digital engagement in the COVID-19 era.

Noteworthy trends emerged in smoking and alcohol consumption, with a percentage of respondents initiating these behaviors during COVID-19. According to this study, 3.9 and 10.8% of respondents, respectively, began smoking and consuming alcohol after COVID-19. Previous research found that during COVID-19, higher rates of alcohol and tobacco use were associated with depressive symptoms, sadness, financial hardship, future uncertainty, and stress (14–16). Remarkably, this study shows that 34.9% of alcohol consumers reported reducing their alcohol use since COVID-19, which is consistent with findings from previous studies (17). The prohibition of social gatherings during the pandemic may be one of the causes. In addition to closing bars and other establishments that served alcohol, the social gathering ban prohibited interacting socially with friends, family, or coworkers (18). Nonetheless, 61.3% of those who drank alcohol stated they had no intention of quitting. As Malaysia navigates the post-pandemic landscape, the insights from this study are critical for informing targeted health interventions. The data showing changes in smoking and alcohol consumption patterns, with some individuals starting these behaviors during the pandemic and notable intentions to quit smoking, underscore the need for focused support services. Tailoring interventions to address these specific behaviors can enhance their effectiveness, improving public health outcomes.

Furthermore, compared to 32.5% of smokers who had the intention but made no attempts to quit, nearly half (42.9%) of current smokers had the intention to quit and had tried to quit. There is evidence that the COVID-19 pandemic would influence smokers’ attitudes toward giving up. According to a survey conducted in the US, most smokers cut back on their use because they intended to stop smoking (19). Health concerns associated with COVID-19 could be used to highlight the dangers of smoking because they could make cessation efforts more successful in persuading individuals to give up the habit during the pandemic (20). Thus, this study sheds light on intentions to quit smoking, influenced by COVID-19-related health concerns, presenting an opportunity for targeted intervention strategies.

Regarding sleeping patterns, the proportion of respondents who slept for less than 7 hours during the pandemic increased compared to before. On the other hand, a few studies found that people slept longer during the pandemic. However, along with the extended period came a shift in sleep schedule and a deterioration in sleep quality (21). Changes in wakefulness and sleep patterns can majorly impact one’s health. In addition to increasing the risk of diabetes, cardiovascular disease, and mental health issues, it has been shown that getting too little sleep is linked to poorer cognitive function and diminished physical performance (22). However, almost half of the study’s respondents said their wake and bedtimes remained the same. Our findings contradicted those of a prior study (23), which documented a significant shift in sleep timing during COVID-19, with respondents staying longer and going to bed earlier. This discussion critically examines the nuanced shifts in health behaviors amid the pandemic, providing valuable insights into the complex interplay of individual choices and external factors during the global health crisis.

The publication of this manuscript is critically important now as it is essential for guiding post-pandemic health interventions, policies, and public awareness efforts. It offers an overview of the change in health behaviors among Malaysians during the pandemic. For example, by understanding how the pandemic has specifically altered health behaviors, individuals can make more informed decisions regarding their health. This knowledge can also foster community support for adopting healthier lifestyles during recovery. Not only that, this study contributes to the global body of knowledge, providing valuable comparative data for researchers and public health officials worldwide. It highlights Malaysia’s unique challenges and responses, offering lessons that can inform global health strategies. Understanding and addressing these shifts will help Malaysia and other countries better navigate the post-pandemic landscape, promoting overall well-being and resilience against future public health challenges.

## Limitation and recommendation

The study’s results have notable limitations that warrant further discussion. Primarily, the cross-sectional design captures health behaviors before and during the pandemic at a single point in time. This raises concerns about the accuracy of participants’ recollections, particularly given the varying stress levels they may have experienced during the pandemic. Although the study acknowledges the potential for recall bias, this significant limitation deserves more in-depth consideration, as the validity of the findings heavily relies on how accurately individuals can recall their health behaviors during such a tumultuous period.

As societies continue to adapt to the ongoing presence of COVID-19, understanding shifts in health behaviors remains crucial. The findings provide valuable insights into how adult Malaysians’ health behaviors changed during the pandemic, but they also raise critical questions about the sustainability of these changes moving forward. In 2024, many individuals may be navigating a “new normal” where previous adaptations could either persist or revert to pre-pandemic patterns. The absence of data on current (post-pandemic) health behaviors complicates our understanding of whether the observed changes are lasting or merely temporary responses to the pandemic. Given that health behaviors may have continued to evolve since data collection in 2022, future research should adopt longitudinal approaches to effectively capture these dynamics over time.

To address these considerations, the implications of this study in the current post-pandemic context could be articulated more clearly. While the findings contribute valuable insights into health behavior changes during the pandemic, their relevance to present-day public health interventions and policies may be limited. However, it is essential for policymakers and public health professionals to recognize the long-term impacts of the COVID-19 pandemic on community health and well-being. By understanding how past behaviors were influenced by the pandemic, strategies can be developed that not only address current health needs but also promote sustained healthy practices.

The study emphasizes the importance of considering the long-term effects of the COVID-19 pandemic and enhancing health promotion strategies that effectively engage the population in positive health behaviors. Leveraging approaches such as “nudges,” as introduced by Thaler (24), could play a key role in encouraging adherence to health-enhancing behaviors (25–30). By implementing subtle interventions that influence decision-making without restricting choices, public health efforts can foster lasting behavior change. Overall, a comprehensive understanding of health behavior trends in the evolving post-pandemic landscape will be essential for guiding future interventions and improving community health.

## Conclusion

The study’s conclusions show notable variations in the respondents’ health-related behaviors before and during the COVID-19 pandemic. There were positive changes in the amount of fruit and plain water consumed daily and the intention to stop smoking. On the other hand, detrimental alterations included decreased physical activity, more screen time, frequent snacking, extended sitting and lying down, and shorter sleep duration.

Maintaining healthy behaviors and changing harmful ones is vital to fend off pandemics and forestall diseases in the future. Therefore, public education is necessary to promote healthy lifestyles actively. The general well-being and resilience of the population can be protected in advance of any future health issues by assisting people in forming and maintaining healthy lifestyles.

## Data Availability

The raw data supporting the conclusions of this article will be made available by the authors, without undue reservation.
